# THE EFFECT OF ADAPTED SPORTS IN QUALITY OF LIFE AND BIOPSYCHOSOCIAL PROFILE OF CHILDREN AND ADOLESCENTS WITH CEREBRAL PALSY

**DOI:** 10.1590/1984-0462/;2017;35;4;00001

**Published:** 2017

**Authors:** Luzanira Correia Feitosa, Sandra Regina Baggio Muzzolon, Danielle Caldas Bufara Rodrigues, Ana Chrystina de Souza Crippa, Marise Bueno Zonta

**Affiliations:** a Universidade Federal do Paraná, Curitiba, PR, Brasil.

**Keywords:** Cerebral palsy, Children/adolescents, Sports, Quality of life, Biopsychosocial profile, Paralisia cerebral, Esporte, Crianças/adolescentes, Qualidade de vida, Perfil biopsicossocial

## Abstract

**Objective:**

The participation in sports and recreational activities promotes inclusion and the quality of life (QOL) for people with some type of disability. This study aims to evaluate and describe the effect of adapted sports (AS) on the QOL and biopsychosocial profile of children/adolescents with cerebral palsy (CP).

**Methods:**

Forty-seven children/adolescents with CP were evaluated and referred to AS (soccer and swimming). The QOL was evaluated by the Pediatric Outcome Data Collection Instrument (PODCI) and the biopsychosocial profile by the Behavior Checklist for Children/Adolescents (CBCL). These instruments considered the influence of gender, age, race, social income, education and topography of spasticity.

**Results:**

Seventeen children/adolescents who practiced AS were re-evaluated after one year. There was significant improvement in the dimensions of *transfers and mobility* (p=0.009), *upper extremity function* (p=0.021) and *global function* (p=0.004) of IARRP. There was significant improvement considering the *attention disorder* syndrome (p=0.026), and the *attention deficit hyperactivity disorders* (p=0.008) in the Diagnostic and Statistical Manual of Mental Disorders (DSM)-oriented analysis (CBCL). Children/adolescents with diplegia obtained greater benefit than those with hemiplegia in relation to the *comfort and pain* (p=0.02) and *global dimension* (p=0.027) (PODCI). The boys had higher scores in *total competence* (p=0.048); the extremely poor group obtained higher levels in the *breaking rules* syndrome (p=0.008).

**Conclusions:**

The AS had a positive effect on the QOL and biopsychosocial profile of children/adolescents with CP in this sample, especially considering the *global* and *upper extremity function*, capacity for *transfers and mobility*, and benefits in the problems related to difficulties in attention.

## RESUMO

**Objetivo:**

A participação em atividades esportivas e recreativas promove a inclusão e a qualidade de vida (QV) de crianças/adolescentes com deficiência. Este estudo visa avaliar e descrever o efeito do esporte adaptado (EA) na QV e o perfil biopsicossocial de crianças/adolescentes com paralisia cerebral (PC).

**Métodos:**

Foram avaliados e encaminhados ao EA (futebol e natação) 47 crianças e adolescentes com PC. A QV foi avaliada pelo Instrumento para Avaliação de Resultados de Reabilitação em Pediatria (IARPP) e o perfil biopsicossocial pela Lista de Verificação Comportamental para Crianças/Adolescentes (CBCL). Foram verificadas as influências de sexo, idade, raça, renda, escolaridade e topografia da espasticidade.

**Resultados:**

Dezessete crianças/adolescentes praticaram o EA e foram reavaliados após um ano. Foi observada melhora significativa nas dimensões *transferências e mobilidade* (*p*=0,009), *função e extremidade superior* (*p*=0,021) e *função global* (*p*=0,004) do IARRP. Houve melhora significativa considerando as síndromes *problemas de atenção* (*p*=0,026) e *problemas de déficit de atenção e hiperatividade* (*p*=0,008) na análise do Manual Diagnóstico e Estatístico de Transtornos Mentais (DSM) Orientado (CBCL). Crianças com diplegia obtiveram mais benefício que aquelas com hemiplegia em relação às dimensões *dor e conforto* (*p*=0,02) e *dimensão global* (*p*=0,027) (IARPP). Os meninos apresentaram maiores escores em *total de competência* (*p*=0,048); o grupo extremamente pobre obteve maiores índices na síndrome *quebrar regras* (*p*=0,008).

**Conclusão:**

O EA apresentou efeito positivo na QV e no perfil biopsicossocial das crianças e dos adolescentes com PC dessa amostra, especialmente considerando: *função global* e de *extremidades superiores*, capacidade para *transferências e mobilidade*, e benefícios nos problemas relacionados às dificuldades na atenção.

## INTRODUCTION

The participation of children/adolescents with some type of disorder in sports and recreational activities has been an important ally for the promotion of quality of life (QOL).^1^ ^,^ ^2^ Besides increasing the physical capacity, minimizing the lack of condition and promoting inclusion,^1^ ^,^ ^2^ the sport is associated with the reduction of the maladaptive behavior and the improvement of self-esteem and social competence in children with disabilities.^3^

The American Academy of Pediatrics recommends the promotion of participation in sports and recreational activities, and warns the parents and pediatricians that they can overestimate the risks or ignore the benefits of physical activity in children with disability.^1^

Several scientific analyses show evidence of the positive influence of physical exercises in behavioral function and mental health.^2^ ^,^ ^3^ ^,^ ^4^ The voluntary, pleasant and moderate practice is associated with indicators of improvement in the mood, cognition, anxiety, and, consequently, the QOL if healthy individuals.^5^

Despite these benefits, few studies have assessed the effect of sports for children/adolescents with impairment in our field, especially for those with cerebral palsy (CP), considered as the most frequent condition of motor incapacity in childhood.^6^ CP causes limitation in the activities, which can be observed in the lower involvement of these individuals in the life in community, in sports and recreational activities.^1^ The model proposed by the World Health Organization (WHO) for the International Classification of Function, Disability and Health prioritizes function as a component of health, and emphasizes that the objectives and results of the interventions proposed to these children should be related with social activity and participation.^7^

Several modalities of adapted sports (AS) are regulated in Brazil, but the access of people with different types of disabilities is still restricted to large urban centers. The National Association of Sports for the Disabled (ANDE) gathers all sports practiced by all types of disability, and coordinates the classification of athletes in respective modalities and according to his or her disability (http://www.ande.org.br/).

The objectives of this study are to assess and describe the effect of AS on the QOL and the biopsychosocial profile of children and adolescents with CP.

## METHOD

Prospective study that assessed the effect of AS in children/adolescents with CP, assisted in the Outpatient Clinic of Spasticity in Pediatrics from the Neuropediatrics Center of Hospital de Clínicas, Universidade Federal do Paraná (UPFR), from August 2011 to August 2015. The study was authorized by the Research Ethics Committee of Hospital das Clínicas in UFPR (CAAE 178.0.208.000-11), and the parents signed the informed consent form, authorizing the participation of their children.

The analysis included children and adolescents of both genders, aged between 6 and 18 years, with functional level I or II, who had specific advisement and practiced AS, responding to the instruments of evaluation before and one year after this activity. The functional level was classified according to the Gross Motor Function Classification System - GMFCS).^8^ The levels of classification I and II characterize higher functional independence. For those in level I, motor function is close to normal, with mild difficulty in coordination and balance, and for those in level II, the difficulty presents itself while jumping or running.^8^

The study excluded children/adolescents who were referred to the practice of AS, but did not respond to the instruments of evaluation. The modalities of AS available were swimming and seven a side soccer, and the children and adolescents in this sample could choose to participate in one or both modalities, determining the number of times in the week they would attend this activity, according to their interest, conditions of access and time available. Swimming approaches different styles, according to the participants’ skills. The adaptation of the seven a side soccer refers to the number of players on each team.

The QOL was assessed by the Instrument for the Evaluation of Results in Pediatrics Rehabilitation (IARRP), adapted from the Pediatric Outcomes Data Collection Instrument (PODCI), from the Pediatric Orthopaedic Society of North America.^9^ ^,^ ^10^ This instrument is composed of 108 questions in six domains: physical function and upper extremity, basic transfer and mobility, physical and sports function, pain and comfort, happiness and global function, and symptoms. The version used in this study is that responded by the parents/tutors of the children and adolescents. The instrument was translated to Portuguese, and then back to English, by a native speaker; then, both versions in English were compared by a judge.^10^ The scores were obtained busing a specific software available in the American Academy of Orthopaedic Surgeons (www.aaos.org). The software provides the normatized and standardized scores, being the latter used for statistical analysis.

The biopsychosocial profile was assessed by the Child Behavior Checklist for ages 6-18 - CBCL), instrument validated in Brazil and used in more than 80 countries.^11^ CBCL is a comprehensive instrument composed of two parts. The first one has 2 items and evaluates the social competence related with the activities, sociability and schooling; and the second one has 118 items, evaluating the existence of emotional and behavioral problems.

It was possible to verify the influence of gender, age, race, income, schooling and topography in the spasticity of QOL and in the biopsychosocial profile. Concerning the income, the following was considered: financial assistance from the government, per capita income and classification 1 for those extremely poor, and 2 for people from the low, middle and higher middle class and lower high class, based on the criteria from the Secretariat of Strategic Topics of the federal government.

The results of quantitative variables were described by means, medians, minimum values, maximum values and standard deviation. The qualitative variables were described by frequency and percentage rates. To compare the pre and post-score evaluations of the studied group, the Wilcoxon test was applied. The Mann-Whitney test was used to compare the groups according to gender, number of activities per week and topographic classification of deficit. P values <0,05 indicated statistical significance. The data were analyzed using the Statistical Package for the Social Sciences (SPSS) (IBM SPSS *Statistics for Windows*, *Version* 20.0, Armonk, New York, United States).

## RESULTS

In the period considered in this study, 46 children/adolescents with CP were referred to AS and, in that occasion, completed the instruments of evaluation proposed in this article. Of these individuals, this study considered 17 children/adolescents who practiced AS, who were reassessed after one year. In the beginning of the study, mean age was 10.6±1.7 years, ranging from 7 to 14 years, and 12 (70.6%) of the participants were male. The functional level of 16 (94%) children was classified as GMFCS I, and only one participants fit in level II. Of the 17 participants analyzed: 11 (64.7%) were hemiplegic and 6 (35.3%) were diplegic. Regarding the modality and the frequency of practice of AS, 11 (64.7%) practiced soccer, 4 (23.5%), swimming, and 2 (11.8%), soccer and swimming - 11 (64.7%) practiced AS once a week. Regarding income, 8 (47%) were considered extremely poor, and 5 (30%) were financially supported by the government. Detailed clinical and identification data of each participant are available in Table 1.

**Table 1:** Clinical characteristics of 17 patients with cerebral palsy who practiced one year of adapted sports.

	Age	Gender	Race	Schooling	Income	Social benefit	GMFCS	Topography	AS modality	Weekly frequency
01	08	Male	White	ES (1st to 5th grade)	2	No	I	Diplegia	Soccer	1
02	10	Male	Brown	ES (1st to 5th grade)	1	No	I	Hemiplegia	Soccer	1
03	09	Male	White	Special class	2	No	I	Hemiplegia	Soccer	1
04	12	Male	White	ES (6th to 9th grade)	2	No	II	Diplegia	Swimming	2
05	10	Male	Brown	ES (1st to 5th grade)	2	Yes	I	Diplegia	Both	3
06	11	Female	White	ES (1st to 5th grade)	2	No	I	Hemiplegia	Soccer	1
07	11	Male	Brown	ES (1st to 5th grade)	1	No	I	Hemiplegia	Soccer	1
08	12	Male	White	ES (6th to 9th grade)	2	No	I	Hemiplegia	Soccer	1
09	10	Male	Black	ES (1st to 5th grade)	2	No	I	Diplegia	Soccer	1
10	10	Male	White	ES (1st to 5th grade)	1	No	I	Hemiplegia	Soccer	1
11	07	Male	White	ES (1st to 5th grade)	2	No	I	Diplegia	Soccer	1
12	14	Male	White	ES (6th to 9th grade)	1	No	I	Diplegia	Soccer	1
13	11	Female	White	ES (1st to 5th grade)	1	No	I	Hemiplegia	Soccer	1
14	11	Female	White	ES (1st to 5th grade)	2	No	I	Hemiplegia	Swimming	2
15	12	Female	White	ES (1st to 5th grade)	1	No	I	Hemiplegia	Swimming	2
16	12	Female	White	Special class	1	No	I	Hemiplegia	Swimming	2
17	11	Male	White	EF (1st to 5th grade)	1	No	I	Hemiplegia	Both	3

AS: adapted sports; ES: Elementary School; INCOME-1: extremely poor, poor, but not extremely poor, and vulnerable; 2: low, middle and high middle class and low high class; GMFCS: Gross Motor Function Classification System.

The increasing scores stood out in the second evaluation of the participants, indicating improvement in the QOL after the practice of AS. There was significant difference in the dimensions transfer and mobility (p=0.009), function and upper extremity (p=0.021) and global function (p=0.004) of IARRP. Likewise, there was an increase in the scores obtained in CBCL after the practice of AS. 

Improvements were observed one year after the practice of sports, with statistically significant difference in the attention disorder syndromes (p=0.026) and the attention deficit hyperactivity disorders (p=0.08), in the analysis of the Diagnostic and Statistical Manual of Mental Disorders (DSM)-oriented, without differences regarding age, race or schooling for any syndrome. The boys presented higher scores of total competence (p=0.048) in CBCL in relation to girls after one year of practicing sports.

Income influenced the behavior of breaking rules: the extremely poor group presented higher score (p=0.008) in this sub-item one after after the practice of sports, indicating more difficulties to follow rules both at home and in school and other environments. There was no difference between hemiplegic and diplegic participants, considering the syndromes in CBCL. In IARRP, it was observed that diplegic participants presented higher mean than hemiplegic participants in the dimension pain and comfort (p=0.02) and in the global dimension (p=0.027) (Table 2).

**Table 2:** Influence of spasticity topography on the difference between both evaluations in the Instrument for the Evaluation of Results in Pediatrics Rehabilitation (IARRP) in the 17 patients with cerebral palsy who practiced one year of adapted sports.

IARRP dimensions	Topography	Difference between evaluations (2^**nd**^ - 1^**st**^ )			p-value*
		n	Mean	SD	
Upper extremity	Hemiplegia	11	0.8	4.3	0.808
	Diplegia	6	3.5	7.3	
Transfer and mobility	Hemiplegia	11	3.7	4.4	0.733
	Diplegia	6	5.2	10.2	
Sports	Hemiplegia	11	6.3	11.8	0.591
	Diplegia	6	6.2	12.8	
Pain and comfort	Hemiplegia	11	-1.8	12.4	0.020
	Diplegia	6	17.8	16.9	
Happiness	Hemiplegia	11	4.1	10.7	0.098
	Diplegia	6	-1.7	4.1	
Global	Hemiplegia	11	2.3	3.6	0.027
	Diplegia	6	8.2	6.1	

*Mann-Whitney nonparametric test, *p*<0.05; SD: standard deviation; n: number of participants.

The mean number of participants who practiced AS once a week was higher than of those who practiced it twice or three times a week (p=0.027). Participants aged up to 10 years presented higher mean in the dimension sports (p=0.014) of IARRP, considering the difference between both evaluations (second-first), with significant difference, thus indicating higher participation in individuals aged more than 10 years, without differences regarding race or schooling.

## DISCUSSION

This study, which verified the effect of AS on the QOL of children and adolescents with CP, can show the significant improvement in the dimensions transfer and mobility, upper extremity function and global function. The positive effect of AS was also observed in the biopsychosocial profile for the positive influence on the attention disorder syndromes and on the attention deficit hyperactivity disorders.

The improvement observed is extremely relevant, considering that children/adolescents with CP present lower scores of QOL, and that these rates interfere in their biopsychosocial and emotional profile.^12^ Children/adolescents with CP tend to depend more on their parents and to perform fewer daily activities, with lower participation in social and recreational activities.^13^ This study showed that AS had a positive effect not only on functionality, which is seen as a health component, but also on the QOL and on the biopsychosocial profile, major objectives of the interventions.

The questionnaire used to measure the quality of life (PODCI) is considered sensitive to detect change throughout time, so it can assess the clinical evolution and the effect of different interventions. The fact that it was only translated, and not validated to Portuguese, may have been a limitation. However, it is important to mention that the translated version has been used in other studies about quality of life nationally.^10^

AS has been associated with improved attention, considered as one of the basic requirements for coordination and motor control. The lack or deficit of attention has a negative impact on the learning of language, writing and motor skills.^14^ The study by Piek *et al.* assessing children with mild central nervous disorders showed that attention significantly affects fine and global motor control performance.^15^

The improvement observed in this sample regarding attention deficit and hyperactivity disorders corroborates several studies that observed the benefits of practicing physical activity for the treatment of the attention deficit hyperactivity disorder, especially concerning the executive functions, such as problem solving, planning and execution of tasks; and for the use and strengthening of the work memory.^16^

Two AS modalities, swimming and seven a side soccer, were available for children and adolescents in this study. Eleven individuals were male, which may explain the higher frequency of those interested in soccer. Only four participants chose swimming, and two practiced seven a side soccer and swimming, which did not allow the separate analysis of the effect of each modality. Eleven (64.7%) participants practiced AS once a week.

The practice of the necessary skills to swim or play soccer promotes benefits in motor coordination, in strength, balance, flexibility, speed and physical conditioning, which may explain the gain observed in the dimensions transfer and mobility, upper extremity function and global function of PODCI.

Dimitrijević *et al.* Investigated the effect of an intensive swimming program on gross motor function and on the water skills of children with CP. After six weeks, the authors observed significant improvement on the motor function, which shows that these children can improve the gross motor function on the ground by practicing in the water.^17^

For Tsutsumi *et al*., the contact with the water environment allows the change of actions and characteristics of the body and its skill levels, regulating and adjusting the coordination of space, speed and strength continuously.^18^ The floating characteristic provided by the water leads individuals with CP to reduce the load and the impact on their joints, and reduces the negative influence of lack of balance and poor postural control, giving them the opportunity to feel their body free of restrictions experienced on the ground.^19^

Soccer, on the other hand, is one of the most practiced sports modalities in the world. This sport requires postural control, and the conduction and handling of the ball with the feet favor balance control, since they require the continuous use of one-foot support.^20^ ^,^ ^21^ This type of activity provides benefits for patients with CP, who are usually accompanied by musculoskeletal and functional limitations, since it promotes flexibility, strength, motor coordination, balance and functional improvement.^22^

In this sample, the boys obtained better scores in relation to girls, which corroborates the study by Azevedo *et al*., which indicated that the practice of moderate and vigorous physical activities was more common among men.^23^ Evidence shows the clear preference of sports by the male gender, especially soccer. Among girls, activities related to walking and dancing are very common.^24^ According to Oehlschlaeger *et al*., in the past decades, men have practiced more systematic physical and sports activities than women. These authors associated these findings with other studies conducted in Brazil, which show the same difference between genders, both in adolescence and in adulthood.^25^ These data point to the need of stimulating the practice of sports among girls, especially those with CP.

Unlike what was observed by other authors, this sample did not show a relationship between lower income and less participation in sports.^26^ On the other hand, it was observed that the behavior of breaking rules was more common among extremely poor individuals. The CBCL, instrument used in this study, evaluates the presence of internalizing problems, such as signs of anxiety and depression, and externalizing issues, such as breaking rules and aggressiveness. The relationship between the breaking rule behavior and socioeconomic condition has been observed in the study by Costello et al*.* ^27^ Besides confirming the relationship between poverty and the presence of mental disorders in children, the authors noticed that the improvement in the socioeconomic condition reduced the externalizing factors; however, it did not interfere in the internalizing ones.

This study pointed out that children with diplegia obtained more benefits than those with hemiplegia concerning the dimension pain and comfort, from PODCI. Considering that all children and adolescents in the sample presented with functional levels I or II, which characterize more independence, this result may be related with the severity of motor involvement. Children/adolescents with hemiplegia presented more functionality and more favorable motor prognosis in comparison to diplegic and quadriplegic individuals,^28^ which might explain the greater benefit regarding pain and comfort for the diplegic participants in this sample. In hemiplegia, the difficulty regarding global balance is lower than the other types of CP. This happens because, unlike diplegia, which is characterized by the impairment of both lower limbs, the sensorimotor system of one of the sides of children with hemiplegia remains relatively intact.^29^

Unlike the expectation, those who practiced AS once a week obtained higher scores than those who took part more often. Maybe this data is related with the sample characteristics, in which 11 (65%) participants practiced AS only once. On the other hand, it may be an important indicator that the practice itself is more relevant than its frequency.

The observation of the greatest participation of children aged more than 10 years may also be related with the sample characteristics - only three children were younger than 10. The age factor interferes significantly in the functional capacity of individuals with CP, especially when considering the musculoskeletal system. CP is a non-progressive change, but the evolution of its effects does not stop between the ages of 16 and 18. Even if it is considered as an incapacitating childhood condition, its effects can be verified in the functional loss that takes place throughout adulthood, which should be considered in the indication of the different interventions.^30^ The inclusion of practices such as sports can stimulate the maintenance of physical activities throughout life, promoting not only functional and motor gain, but also an improved QOL.

The referral of children/adolescents with CP is still limited in our field. In this study, the invitation to participate in AS was presented to 46 children/adolescents during their routine outpatient appointment. Different reasons are associated with the low adherence observed, and only 17 individuals took part in the proposed activity, being therefore included in the study. The fact that, among these, 16 were in functional level I suggests that the functional limitations could have been one of these reasons. According to Murphy, functional limitation is one of the main barriers to the participation in sports activities, followed by costs and difficulties of access.^1^ The fact that the sample is small and mostly composed of GMFCS I may be considered as a limitation of this study. For the American Academy of Pediatrics, children with disabilities have a more restricted participation in sports and recreational activities. The institution emphasizes the need to identify these barriers in the local, state and federal contexts, aiming at including all children with disabilities in appropriate activities.^1^

A recent study by Gordia *et al*. Concluded that the specific knowledge for the recommendation of physical activity is limited among pediatricians, considering the recommendation for typical chidlren/adolescents.^31^ There are no studies in Brazil about the recommendation of physical or sports activities for children/adolescents with CP, but results such as the ones in this study are important to warn professionals about the importance of this practice, in order to stimulate new investigations.

Finally, the AS promoted benefits in the QOL of children and adolescents with CP in this sample, especially in the dimensions transfer and mobility, upper extremity function and global function. There was also a significant improvement in the biopsychosocial profile, considering the attention disorder syndrome and the attention hyperactivity deficit disorder in CBCL.
